# Association of non-alcoholic fatty liver disease with self-reported osteoarthritis among the US adults

**DOI:** 10.1186/s13075-024-03272-2

**Published:** 2024-01-31

**Authors:** Yu Lu, Jianyu Zhang, Hejun Li, Ting Li

**Affiliations:** Department of Rheumatology and Immunology, Guangdong Provincial Key Laboratory of Major Obstetric Diseases, Guangdong Provincial Clinical Research Center for Obstetrics and Gynecology, The Third Affiliated Hospital of Guangzhou Medical University, Guangzhou, China

**Keywords:** Osteoarthritis, NAFLD, NHANES, Cross-sectional, United States

## Abstract

**Background:**

The association between non-alcoholic fatty liver disease (NAFLD) and osteoarthritis (OA) has not been well elucidated. The aim of the present study was to investigate the association between NAFLD and OA in the US adults.

**Methods:**

A cross-sectional study was performed on participants in the 2017–2018 National Health and Nutrition Examination Survey (NHANES) cycle. NAFLD was defined by the vibration-controlled transient elastography. The diagnosis of OA was based on self-reported data. Weighted multiple logistic regression models and stratified analyses were performed to explore the relationship and verify the stability of the conclusions. Sensitivity analysis using multiple imputation for missing data and propensity score matching (PSM) were performed.

**Results:**

In total, 2622 participants [Male: 1260 (47.8%)] were included in this study with a mean age of 48.1 years old (95% CI, 46.6–49.6 years old), containing 317 (12.8%) OA patients and 1140 NAFLD patients (41.5%). A logistic regression indicated a significant association between NAFLD and OA without adjustment [odds ratio (OR) = 2.05; 95% CI, 1.52–2.78]. The association remained stable after adjustment for covariates (OR = 1.72; 95% CI, 1.26–2.34). Sensitivity analysis of missing data with multiple interpolation and PSM found similar results. A significant and consistent association of NAFLD with OA was still observed in each subgroup stratified by age and metabolic syndrome (MetS). Stratified by sex, obesity, and sensitivity c-reactive protein (hs-CRP) category, a statistically significant association was only shown in females, those without obesity, and those without hyper hs-CRP. The results illustrated that the relationship between NAFLD and OA was stable in all subgroups and had no interaction.

**Conclusions:**

NAFLD was positively correlated with OA. Given the current pandemic of NAFLD and OA, clinicians should screen for NAFLD in arthritis patients and intervene early.

## Introduction

Fueled by population aging, osteoarthritis (OA) has become the most prevalent and disabling degenerative joint disease, estimated to affect at least 36 million United States (US) adults and imposing a huge disease burden[1]. In recent years, with the deepening of research on OA, researchers tend to view OA as a complex multiple-factor disease rather than a representative degenerative disease[2]. Various factors, such as obesity, low-grade inflammation, and insulin resistance (IR), seem to have a crucial effect on the pathogenesis of OA[2, 3]. Accumulating evidence has indicated that OA was associated with several systemic diseases such as obesity, cardiovascular disease, type 2 diabetes mellitus (T2DM), and metabolic syndrome (MetS) [4–6]. Besides, current treatments for OA have limited effectiveness, mainly focusing on relieving symptoms rather than preventing or reversing the progression of the disease[7, 8]. Therefore, identification of risk factors for OA and intervention may reduce the disease burden of OA.

Non-alcoholic fatty liver disease (NAFLD) is characterized by the presence of ≥ 5% hepatic steatosis caused by viral hepatitis, excessive alcohol consumption, and drugs[9]. With a global prevalence of 32.4% which is projected to increase for the next decade, NAFLD has become a major global public health problem[10]. In fact, even excluding social costs, annual direct medical costs directly attributable to NAFLD in the USA are estimated to exceed $100 billion[11]. The mechanism of NAFLD is multi-factorial involving IR and metabolic disorders that trigger low-grade inflammation in the liver and extra-hepatic organs[12]. Considering its close association with metabolic dysfunction, a growing body of evidence in the literature suggests that NAFLD is the hepatic manifestation of MetS[13].

Mechanistically, low-grade inflammation and metabolic derangements may at least be involved in the occurrence of NAFLD and OA[2, 14]. It means that these two diseases have similar causative factors. However, few studies have explored the correlation between NAFLD and OA[15]. Hence, in this cross-sectional study, we used the data from the 2017–2018 National Health and Nutrition Examination Survey (NHANES) cycle to explore the potential association between NAFLD and OA.

## Methods

### Study design

The NHANES is a nationally representative continuity program conducted by the National Center for Health Statistics (NCHS), part of the Centers for Disease Control and Prevention (CDC), that focuses on a variety of health and nutrition measurements. The program surveys the health and nutrient status of the general non-institutionalized civilian population of the USA every 2 years by using a stratified, multistage, clustered probability sampling method. The data used in the presented study were all obtained from the 2017–2018 cycle of the NHANES. The survey was approved by the Research Ethics Review Committee of the CDC, which was approved by all adult participants in written informed consent. The third affiliated hospital of Guangzhou Medical University determined that the presented study was exempt from review because all personal information in the datasets used in the analysis were fully de-identified.

**Fig. 1:**
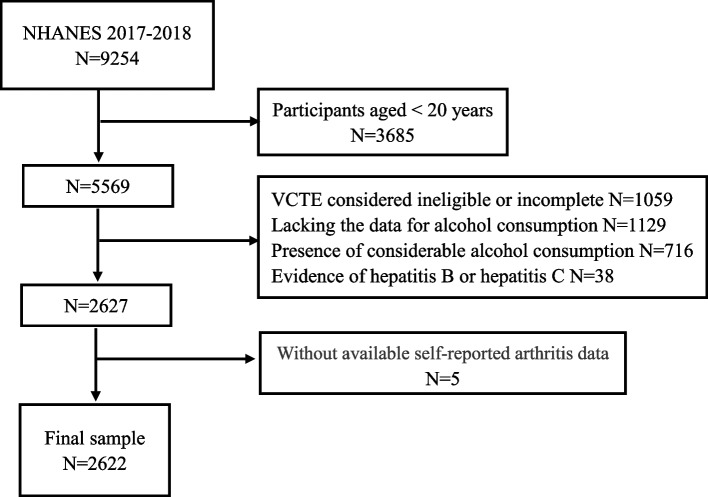
Flow chart of the study participants

Initially, 9254 participants from the 2017–2018 cycle of the NHANES were included in the presented study. Those aged less than 20 years old were excluded (*n* = 3685). Of the 5569 participants, 2942 were eliminated because the presence of one of four conditions: ineligible vibration-controlled transient elastography (VCTE) assessment or an incomplete VCTE exam (*n* = 1059), lack of data for alcohol consumption (*n* = 1129), considerable alcohol consumption (*n* = 716), history of hepatitis B (*n* = 17) or hepatitis C (*n* = 21). Finally, we excluded patients without available self-reported arthritis data (*n* = 5), leaving 2262 individuals for the final analysis (Fig. 1).

### Diagnosis of NAFLD by VCTE

Despite the fact that liver tissue biopsy evaluation is recommended by clinical guidelines as the golden standard for diagnosing hepatic disease, it is impractical to perform liver biopsy examination to assess hepatic steatosis for large populations, given the current global prevalence of patients with NAFLD [9, 16, 17]. VCTE is a widely used non-invasive and convenient method to identify hepatic steatosis through the value of controlled attenuation parameter (CAP)[18]. In the NHANES 2017–2018 cycle, VCTE was conducted in the Mobile Examination Center (MEC) using the FibroScan®502 V2 Touch instrument. In the presented study, individuals were diagnosed with hepatic steatosis by CAP ≥ 274 dB/m, as this threshold showed high accuracy in identifying hepatic steatosis[19]. More than 21 drinks/week for men and more than 14 drinks/week for women was considered considerable alcohol consumption[9]. Viral hepatitis was defined as positive for serum hepatitis B surface antigen (HBsAg) test or serum hepatitis C antibody test.

### Diagnosis of osteoarthritis

In epidemiological studies, self-reported OA is often used for case definition[20]. March et al. showed up to 81% agreement between self-reported OA on the questionnaire and clinically defined OA[21]. The NHANES survey asked patients if they had ever been told by a doctor or other healthcare professionals that they had arthritis. If the respondents’ answer was “yes,” they were defined as arthritis. Subsequently, OA patients were identified by answering “Osteoarthritis” to the question “Which type of arthritis was it?”.

### Covariates

Covariables in the presented study included age, sex, race, educational level, marital status, family poverty income ratio (PIR), height, weight, waist circumference, body mass index (BMI), high sensitivity c-reactive protein (hs-CRP), glycated hemoglobin A1c (HbA1c), fasting blood glucose (FBG), triglyceride (TG), total cholesterol (TC), high-density lipoprotein cholesterol (HDL-c), low-density lipoprotein cholesterol (LDL-c), uric acid, alanine aminotransferase (ALT), aspartate aminotransferase (AST), γ-glutamyl transpeptidase (GGT), systolic blood pressure (SBP), diastolic blood pressure (DBP), diabetes, MetS, smoking status and drinking consumption. The demographic information included age, sex (male, female), race, educational level (high school and below, some college and above), marital status (living with partner, single) and PIR [low income (≤ 1.3), middle income (> 1.3 to 3.5) and high income (≥ 3.5)][22]. The anthropometric data included height (cm), weight (kg), and waist circumference (cm). BMI (kg/m^2^) was calculated as survey-measured weight (kg) in kilograms divided by height (m^2^). The laboratory data included HbA1c (%), FBG (mg/dL), TG (mg/dL), TC (mg/dL), HDL-c (mg/dL), LDL-c (mg/dL), uric acid (mg/dL), ALT (U/L), AST (U/L), and GGT (U/L). Besides, hs-CRP (mg/dL) status is defined according to clinically recommended high-risk thresholds for hs-CRP[23].

Diabetes mellitus was determined by FBG ≥ 126 mg/dL or HbA1c level ≥ 6.5%, self-reported diabetes, or administration of anti-diabetic drugs (including insulin) [24]. Blood pressure was measured through oscillography protocols by health technicians who were certified for BP measurement through a strict training program. After sitting quietly for 5 min, the participants had their blood pressure (systolic and diastolic) measured three times at 60-s intervals using Omron HEM–907XL. Therefore, the mean value of three oscillometric readings of the sphygmomanometer was used as the blood pressure of the participants in this study. Hypertension was determined by SBP ≥ 140 mmHg and/or DBP ≥ 90 mmHg or self-reported current use of antihypertensive drugs[25]. MetS was diagnosed in accordance with the National Cholesterol Education Program Adult Treatment Panel III guidelines[26].

The smoking status was categorized as current smoker (had smoked ≥ 100 cigarettes in their lifetime and smoking everyday/somedays), former smoker (had smoked ≥ 100 cigarettes in their lifetime but not smoking now), and never smoker (had smoked < 100 cigarettes in their lifetime). Alcohol consumption was determined by the survey question: “In any one year, had at least 12 drinks of any type of alcoholic beverage?” The participants were divided into alcoholic drinkers groups or non-alcoholic drinkers groups.

### Statistical analysis

Given the stratified multi-stage probabilistic sampling design used by the NHANES, we utilized weights recommended by the NHANES website and reporting guidance in our statistical analyses to ensure that the datasets used in the present study were as representative as possible of the entire general non-institutionalized population in the USA. Continuous and classified variables were presented as weighted numbers (weighted percentage) (95% CI). For comparison between groups, the chi-square test was used to compare categorical variable data and the Kruskal–Wallis test to compare continuous variables. A weighted multivariable logistic regression model was conducted to estimate odds ratios (ORs) and 95% confidence intervals (Cis) to describe the association between NAFLD and OA. Four weighted logistic regression models were constructed to account for the influence of covariates. Model 1 was unadjusted. Model 2 was adjusted for sex, age, race, educational level, marital status, and PIR. Model 3 was based on Model 2 and adjusted for drinking status and smoking status. The fully adjusted model (model 4) was based on Model 3 with hyper hs-CRP and MetS adjusted. Besides, we conducted the subgroup and interaction analyses starfield by age group (< 60 years old, ≥ 60 years old), sex, hs-CRP category, obesity and MetS using weighted multivariable logistic regression models. To assess the robustness of our findings, we performed several sensitivity analyses. Propensity score matching (PSM) was used to balance covariates, including age, with multifactorial logistic regression adjusting for sex, age, race, smoking status, and Mets. Multiple imputation by chained equations (MICE) and repeated the main analyses. We used multiple imputation, based on 5 imputed data sets to account for missing baseline data.

All statistical analyses were conducted by R software (version 4.2.0). All tests were two-tailed and *P* values less than 0.05 were considered statistically significant.

## Results

### Study participants and baseline characteristics

The baseline characteristics of subjects with or without OA were presented in Table 1. A total of 2622 participants (male: 1260 (47.8%)) were included in the present study with a mean age of 48.1 years old (95% CI, 46.6–49.6 years old) based on the weighted analyses. Including 1140 participants (41.5%; 95% CI, 39.3–43.8%) diagnosed as NAFLD, this sample consisted of 317 (12.8%; 95% CI, 10.1–16.1%) patients diagnosed with OA, and 2305 (87.2%; 95% CI, 83.9–89.9%) who did not have OA. Compared to participants without OA, those with OA were more likely to be older, female, Non-Hispanic White, current smokers with diabetics, hypertension, obesity, and MetS. Moreover, these OA participants had a larger waist circumference.

**Table 1:** Baseline characteristics of the study participants

Characteristic	Total (*n* = 2622)	Without osteoarthritis (*n* = 2305)	With osteoarthritis (*n* = 317)	*P* value
Age, years	48.1 (46.6–49.6)	46.0 (44.6–47.4)	62.4 (61.2–63.6)	< 0.01
< 60	1687 (71.7)	1577 (76.5)	110 (39.0)	
≥ 60	935 (28.3)	728 (23.5)	207 (61.0)	
Sex				< 0.01
Male	1260 (47.8)	1152 (50.3)	108 (30.7)	
Female	1362 (52.2)	1153 (49.7)	209 (69.3)	
Race				< 0.01
Mexican American	311 (7.3)	284 (7.81)	27 (3.9)	
Other Hispanic	232 (6.6)	216 (7.2)	16 (2.6)	
Non-Hispanic White	860 (63.6)	693 (61.6)	167 (77.6)	
Non-Hispanic Black	641 (11.8)	584 (12.4)	57 (7.7)	
Other Race	578 (10.6)	528 (11.0)	50 (8.1)	
Educational level				0.26
High school and below	1648 (66.7)	1447 (67.2)	201 (63.4)	
Some college and above	971 (33.3)	855 (32.8)	116 (36.6)	
Married status				0.47
Living with partner	1007 (35.2)	881 (35.6)	126 (33.0)	
Single	1614 (64.8)	1423 (64.4)	191 (67.0)	
PIR				0.65
≤ 1.3	530 (15.7)	480 (16.0)	50 (13.3)	
≥ 1.3 to 3.5	917 (33.2)	798 (33.4)	119 (32.1)	
≥ 3.5	858 (51.1)	745 (50.6)	113 (54.6)	
BMI, kg/m^2^	29.4 (28.9–30.0)	29.2 (28.6–29.8)	31.0 (29.9–32.2)	< 0.01
WC, cm	99.7 (98.5–101.0)	99.0 (97.7–100.4)	104.4 (101.6–107.2)	< 0.01
ALT, U/L	22.6 (22.0–23.3)	22.7 (22.0–23.5)	22.1 (19.6–24.5)	0.66
AST, U/L	21.7 (21.0–22.4)	21.8 (20.9–22.6)	21.1 (19.8–22.4)	0.44
GGT, IU/L	28.0 (26.5–29.5)	28.1 (26.5–29.7)	27.3 (23.9–30.6)	0.66
UA, mg/dl	5.3 (5.3–5.4)	5.4 (5.3–5.4)	5.3 (5.0–5.5)	0.47
hs-CRP, mg/L	3.6 (3.2–3.9)	3.5 (3.1–3.9)	3.9 (3.1–4.7)	0.43
< 2.5	1494 (62.1)	1329 (62.9)	165 (57.3)	
≥ 2.5	976 (37.9)	837 (37.1)	139 (42.7)	
Smoking status				0.02
Never	1685 (65.1)	1509 (66.2)	176 (58.1)	
Former	349 (10.5)	308 (10.8)	41 (9.0)	
Current	588 (24.3)	488 (23.1)	100 (32.9)	
Drinking status				0.66
No	432 (11.7)	393 (11.9)	39 (10.4)	
Yes	2190 (88.3)	1912 (88.10)	278 (89.6)	
Diabetes	494 (14.1)	403 (13.1)	91 (20.8)	0.02
Hypertension	1122 (36.4)	915 (33.3)	207 (58.1)	< 0.01
Obesity	1044 (41.3)	884 (39.6)	160 (52.8)	< 0.01
MetS	476 (16.5)	384 (15.4)	92 (23.6)	0.02
NAFLD	1140 (41.5)	970 (39.3)	170 (57.0)	< 0.01

*Abbreviations: PIR*, family poverty income ratio; *BMI*, body mass index; *WC*, waist circumference; *UA*, uric acid; *MetS*, metabolic syndrome; *NAFLD*, non–alcoholic fatty liver disease

The characteristics of participants are described as means (95% CIs) for continuous variables and unweighted numbers (weighted percentage) for categorical variables

### Multivariable regression analyses

We examined the association between NAFLD and OA in the weighted multivariable logistic regression analysis (Table 2). Without adjusting for any covariates, a significant correlation between NAFLD and OA was detected in Model 1 (OR = 2.05; 95% CI, 1.52–2.78). Moreover, our results showed that the association between NAFLD and OA remained stable and significant even with adjustment for sex, age, race, smoking status, and MetS (OR = 1.72; 95% CI, 1.26–2.34).

**Table 2:** Association of NAFLD with osteoarthritis among participants in the NHANES 2017–2018 cycle

Model	Osteoarthritis		
	OR	(95%CI)	*P*-value
Model 1	2.05	1.52–2.78	0.001
Model 2	1.76	1.26–2.45	0.011
Model 3	1.77	1.23–2.47	0.014
Model 4	1.72	1.26–2.34	0.018

Model 1: adjusted for none

Model 2: adjusted for sex, age, and race

Model 3: adjusted for sex, age, race, and smoking status

Model 4: adjusted for sex, age, race, smoking status, and MetS

### Subgroup analyses

**Fig. 2:**
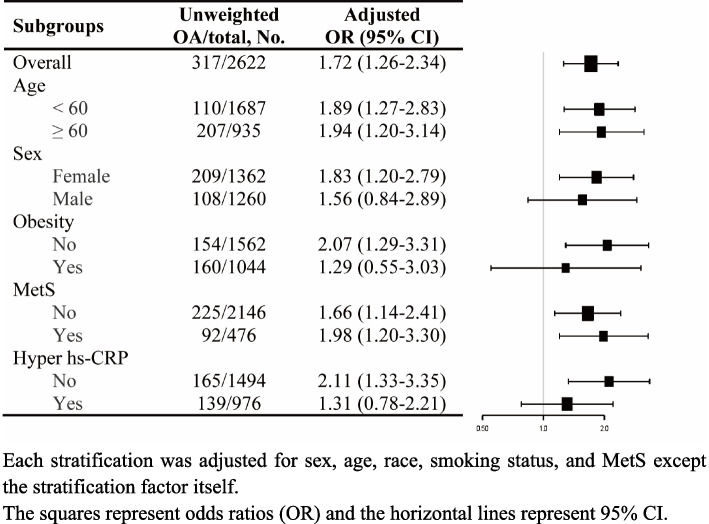
Stratified analyses of the associations between NAFLD and osteoarthritis among participants in the NHANES 2017–2018 cycle

Subsequently, we conducted stratified analysis stratified by age (< 60, ≥ 60), sex, obesity, MetS, and hs-CRP category (< 2.5, ≥ 2.5), and the results of subgroup analysis of NAFLD and OA were presented in Fig. 2. Our subgroup analysis results revealed a significant and consistent association of NAFLD with OA in each subgroup stratified by age and MetS (all *P* < 0.05). When stratified by sex, obesity, hs-CRP category, a statistically significant correlation was only observed in female (OR = 1.83; 95% CI, 1.20–2.79), those without obesity (OR = 2.07; 95% CI, 1.29–3.31), and those without hyper hs-CRP (OR = 2.11; 95% CI, 1.33–3.35). The results illustrated that the relationship between NAFLD and OA was stable in all subgroups and had no interaction (all *P* > 0.05).

### Sensitivity analyses

NAFLD was significantly associated with OA, after PSM: OR = 1.29; 95% CI, 1.02–1.63 (Table 3). Besides, using multiple imputation for baseline missing data, NAFLD was still associated with OA.

**Table 3:** Association of NAFLD with osteoarthritis among participants in the NHANES 2017–2018 cycle after propensity score matching

Model	Osteoarthritis		
	OR	(95%CI)	*P*-value
Model 1	1.31	1.06–1.62	0.014
Model 2	1.40	1.23–1.75	0.003
Model 3	1.43	1.15–1.79	0.002
Model 4	1.29	1.02–1.63	0.031

Model 1: adjusted for none

Model 2: adjusted for sex, age, and race

Model 3: adjusted for sex, age, race, and smoking status

Model 4: adjusted for sex, age, race, smoking status, and MetS

## Discussion

In this data analysis of 2622 participants based on the NHANES 2017–2018 cycle, we found that a higher prevalence of osteoarthritis was observed in participants with NAFLD, independent of confounding factors such as sex, age, race, educational level, marital status, PIR, drinking status, smoking status, hyper hs-CRP, and MetS. Besides, in the stratified analysis, we also found that this association only exists in men and those without obesity or MetS. These results conclude that NAFLD is an independent risk marker for OA, suggesting that further evaluation of the underlying mechanism between NAFLD and increased risk of OA is deserved.

To the best of our knowledge, only one previous cross-sectional study involving 3027 Korean individuals aged 50 years and over has explored the potential association between metabolic dysfunction-related fatty liver disease (MAFLD) and OA[15]. Han found that those individuals with MAFLD had a higher ratio of OA in the multiple logistic regression model, after adjusting for age, sex, educational level, smoking, and alcohol consumption (OR = 1.475; 95% CI, 1.18-1.84). Our conclusions agree with his results[15], and we tend to extrapolate this association to the general population of the USA. However, several limitations should be considered in their research. Firstly, the researchers did not have access to NAFLD diagnoses based on ultrasound, and instead, they used an alternative measure of serum biomarker that can be used to identify hepatic steatosis, the fatty liver index (FLI). Besides, the criteria for diagnosing MAFLD in Han’s study may not be accurate enough due to the lack of hs-CRP data. Finally, although Han’s study included a range of confounding factors in their analysis, it did not include factors such as metabolic or inflammatory indicators that might influence the results. Therefore, our study better answers this research question by addressing these questions and is also the first study to investigate NAFLD and OA in the general adult population of the USA.

Barritt AS et al. confirmed that fueled by the severity of liver disease, the prevalence of OA increased gradually[27]. This finding emphasizes the importance of liver disease, especially its disease status, in OA. Nevertheless, the mechanism between NAFLD and OA has not been conclusively defined, but it can be explained by the following points.

Although obesity-related biomechanical overloading plays a predominant role in OA, it is worth noting that inflammatory mediators are also one of the crucial drivers of joint tissue destruction[28, 29]. Firstly, the inflammatory status may be critical to linking OA and NAFLD. For instance, the activation of adenosine 2A receptors (A2AR) is closely related to the downregulation of OA inflammation[30]. Similarly, A2AR also is involved in NAFLD. In addition to enhancing the pro-inflammatory response, A2AR deficiency can also increase fat deposition of hepatocytes[31]. A preponderance of evidence has illustrated that decreased serum Neuregulin 4 (Nrg4) level is detected in NAFLD patients and it is independently correlated with NAFLD, whilst its deficiency accelerates the process of inflammation, liver injury, and fibrosis, and in NASH mice[32, 33]. In addition, Nrg4 may alleviate the progression of OA by attenuating inflammation (IL-Iβ, IL-6, TNF-α) and protecting chondrocyte apoptosis through the MAPK/JNK signaling pathway[34].

Secondly, NAFLD is the manifestation of complex metabolic dysfunction[13, 35]. A series of metabolic risk factors or diseases, including visceral obesity, IR and dyslipidemia, may at least partially promote the occurrence and development of OA. These metabolic disorders have been linked to OA with causal or observational evidence[36–39].

Thirdly, studies have noted that the leukocyte cell‐derived chemotaxin‐2 (LECT2) level was related to the pathogenesis of OA[40]. Recent evidence in mice demonstrated that dipeptidyl peptidase-4 (DPP-4) inhibitors could improve the degree of hepatic steatosis and IR through AMPK-dependent and JNK-dependent inhibition of LECT2 expression[41].

Last but not least, endoplasmic reticulum (ER) stress may bridge the link between NAFLD and OA. ER stress plays a key role in the pathogenesis and progression of bone and joint diseases, involving ER stress in cartilage degradation, synovitis, meniscal lesions, and subchondral bone remodeling[42]. Besides, evidence supports the notion that the existence of ER stress can trigger the occurrence and progression of a variety of liver diseases, especially NAFLD[43, 44].

There are some limitations in the present study which should be considered. Firstly, given the cross-sectional observational nature of the present study, the observed association between NAFLD and OA cannot be assumed to infer causality. Longitudinal evidence is needed in the future to improve the reliability of the conclusion. Secondly, although we have considered a series of confounding factors and conducted a stratified analysis to clarify the potential impact of the association between NAFLD and OA, it is undeniable that there are still some potential confounding factors that have not been considered. Thirdly, the diagnosis of OA is based solely on self-reported data, which may be subject to recall bias. Nonetheless, previous researches had shown a high degree of consistency in self-reporting of OA.

## Conclusions

By using large-scale cross-sectional data from NHANES, the presented study clarified the relationship between NAFLD and OA. Compared to patients with NAFLD, those without showed a lower likelihood of OA. Given the current pandemic of NAFLD and OA, clinicians should screen for NAFLD in arthritis patients and intervene early.

## Data Availability

Publicly available datasets were analyzed in this study. This data can be found here: The National Health and Nutrition Examination Survey dataset at https://www.cdc.gov/nchs/nhanes/index.htm.

## Abbreviations

NAFLD: Non-alcoholic fatty liver disease
OA: Osteoarthritis
NHANES: National Health and Nutrition Examination Survey
IR: Insulin resistance
T2DM: Type 2 diabetes mellitus
MetS: Metabolic syndrome
